# Superlong‐Range Magnetic Coupling and Ferromagnetic Spin Freezing in Mechanoluminescent Semiconductor Eu:SrAl_2_O_4_

**DOI:** 10.1002/advs.202509474

**Published:** 2025-07-31

**Authors:** Xu‐Guang Zheng, Ichihiro Yamauchi, Tomasz Galica, Eiji Nishibori, Tatsuya Kawae, Jumpei G. Nakamura, Akihiro Koda, Chao‐Nan Xu

**Affiliations:** ^1^ Department of Physics Faculty of Science and Engineering Saga University Saga 840‐8502 Japan; ^2^ Department of Materials Science and Engineering Faculty of Engineering Tohoku University Sendai 980‐8579 Japan; ^3^ Department of Physics Faculty of Pure and Applied Sciences Tsukuba Research Center for Energy Materials Science(TREMS) Hydrogen Boride Research Center (HBRC) Tsukuba Institute for Advanced Research (TIAR) University of Tsukuba Ibaraki 305‐8571 Japan; ^4^ Department of Applied Quantum Physics Faculty of Engineering Kyushu University Fukuoka 819‐0395 Japan; ^5^ Muon Science Laboratory Institute of Materials Structure Science High Energy Accelerator Research Organization Ibaraki 319‐1106 Japan

**Keywords:** bound magnetic polaron, dilute magnetic semiconductor, ferromagnetism, mechanoluminescent material, light radiation effect, oxygen vacancy, superlong‐range magnetic coupling

## Abstract

Magnetic transition in nonmetals requires the presence of a considerable proportion of magnetic spins. A new type of ferromagnet named dilute ferromagnetism that contradicts this well‐established concept is proposed for semiconductors of ZnO etc. but has remained experimentally unproven. In this study, an unconventional superlong‐range magnetic coupling and ferromagnetic spin freezing are reported, which can be viewed as an experimental realization of an intrinsic dilute ferromagnetism, in mechanoluminescent material of Eu*_x_* Sr_1‐_ *_x_* Al_2_O_4_ (*x* = 0.2−2%), wherein Eu is sparsely incorporated into the lattice to substitute Sr. Ferromagnetic coupling appears below ≈80 K and fully saturated ferromagnetic magnetization appears below ≈3 K, with an unusually large magnetic moment of ≈14 µ_B_ per Eu^2+^. Muon spin spectroscopy demonstrates intrinsic spin freezing with a spontaneous internal field developed below *T*_C_ of ≈3 K. The neighboring magnetic Eu^2+^ ions in the lattice have an exceptionally large separation more than one order of magnitude larger than those in conventional magnets, marking it as a unconventional magnetic order over a superlong distance. Bound magnetic polarons arising from electrons trapped at oxygen vacancies may account for this unconventional ferromagnetism. Magnetization under light radiation supports this scenario.

## Graphical abstract

Eu:SrAl_2_O_4_ emit visible light even upon finger contact. This mechanoluminescent material is an optimal platform for bound magnetic polarons to produce unexpected ferromagnetism, with the magnetic spins of Eu^2+^ sparsely distributed in the crystal lattice. With simultaneous magnetism and mechanoluminescence, force‐light‐spin‐electron multiple conversion and control can be realized showing high potential for unprecedented applications.

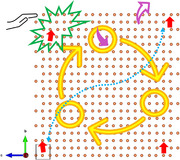

## 1 Introduction

Since the report of a partial ferromagnetic order suspected from the magnetotransport properties of (In_0.987_Mn_0.013_)As^[^ ^1^ ^]^ and a theoretical prediction of room‐temperature ferromagnetism in wide‐gap semiconductor ZnO at the beginning of this century,^[^ ^2^ ^]^ tremendous research has been conducted on this subject. As such ferromagnets have considerable potential for applications in magnetically controllable new spintronics; the origin of their ferromagnetism is also fascinating for condensed matter physics. Ferromagnetic‐like magnetization (i.e., *M*‐*H* behavior) was also observed in the dielectric HfO_2_ thin film without magnetic impurity doping.^[^ ^3^ ^]^ A spin‐polarized positron annihilation spectroscopy study revealed that defects in ZnO could produce ferromagnetic‐like magnetization.^[^ ^4^ ^]^ However, these ferromagnetic‐like behaviors in the so‐called dilute magnetic semiconductors (insulators) were based on magnetization measurements, which are prone to contamination due to a micro quantity of extrinsic ferromagnetic impurities. The study in ref. [4] can be explained by the paramagnetic magnetization of defect‐trapped electrons instead of intrinsic ferromagnetism. In fact, several microscopic investigations of ZnO have shown that the doped magnetic impurities do not enter the lattice, and the previously reported dilute ferromagnetic behaviors in ZnO are primarily due to extrinsic impurities or defects and grain boundaries.^[^ ^5^ ^]^ Our investigations of ZnO have shown that the substitution limit for magnetic ions in ZnO is well below 0.1%, which is far lower than those assumed in previous dilute magnetism reports. We have also found that defect‐trapped electrons in ZnO produce paramagnetic magnetization with saturated *M‐* *H* behavior at low temperatures; however, this is not intrinsic ferromagnetism because the reliable experimental technique of muon spin spectroscopy (µSR), which probes the volume fraction of the magnetic phase in the specimen, does not show any trace of magnetism. More precise theoretical studies in the same scheme of the theoretical model in ref. [2] have refuted most previous predictions of ferromagnetism.^[^ ^6^ ^]^ To date, robust evidence for intrinsic diluted ferromagnetism using reliable experimental methods, such as neutron scattering or µSR, is lacking, thus leading to significant doubts regarding dilute ferromagnetism. In particular, muon has an exceptionally large gyromagnetic ratio of 135.53 MHz T^−1^, making µSR a sensitive magnetic probe that should be optimal for the proposed dilute ferromagnetism.

Theoretically, the presence of dilute ferromagnetism in an intrinsic semiconductor (insulator), wherein possible coupling via conduction electrons is absent, is a significant challenge. According to the universal percolation theory for phase transitions, transitioning a system into a magnetically ordered phase requires a considerable proportion of magnetic spins in the system, such that the spins can connect with their neighboring spins into a long‐range order. For example, the concentration thresholds necessary for this connection in the site percolation model were predicted to be  ≈59% and 31% for a square lattice and a simple cubic lattice, respectively.^[^ ^7^ ^]^ The 50% threshold for neighbor‐4 2D lattice in the bond percolation model, which is considered more appropriate for magnetic exchange couplings than the site percolation model, has been experimentally verified to be exactly valid in the Kagome lattice by our recent research.^[^ ^8^ ^]^ These predicted threshold values far exceed the dilute concentrations. In general, dominating magnetic coupling in insulators is limited to the nearest neighboring atoms with a spacing of no more than ≈5 Å^[^ ^9^ ^]^ due to the fact that the strength of magnetic interactions depends on the overlap integrals between the wave functions of neighboring magnetic ions. Exceptionally long‐range coupling has been reported in metallic systems,^[^ ^10^, ^11^, ^12^ ^]^ where the delocalized electrons serve as the coupling intermediary and can be theoretically explained.^[^ ^13^ ^]^ Therefore, it is strikingly unconventional for the diluted ferromagnetism to appear in a non‐metalic system. Here, we term the magnetic coupling in insulative dilute ferromagnetic materials, in which a typical concentration of ≈1% magnetic ions means a neighboring‐magnetic‐ion separation of more than four chemical bonding of the parent material, as superlong‐range magnetic coupling.

However, when applying µSR to investigate the atomic‐scale mechanism of mechanoluminescence (ML), we unexpectedly observed intrinsic dilute ferromagnetism in a representative ML material, Eu^2+^:SrAl_2_O_4_. This type of mechanoluminescent semiconductor emits visible light upon elastic deformation due to a small mechanical force.^[^ ^14^, ^15^, ^16^, ^17^, ^18^ ^]^ These materials are insulative and feature a micro number of rare‐earth elements such as Eu^2+^ substituted into the lattice, which act as emitting centers for luminescence, and oxygen vacancies that trap electrons for a recombination process to emit ML light (the elastic deformation upon applying a mechanical force is considered to release the trapped electrons to the emitting center to generate ML). Because the bound magnetic polaron (BMP) model has been proposed as the most probable mechanism for dilute ferromagnetism,^[^ ^19^ ^]^ we believe that the presence of abundant defects and the incorporation of magnetic ions into the lattice make ML materials optimum candidates for searching for extant dilute ferromagnetism. The efficient concentration of Eu^2+^ for ML in Eu:SrAl_2_O_4_ is ≈0.2%. In this study, we have found that the substitution limit of Sr by Eu in SrAl_2_O_4_ may be increased to 2−3%. Characteristic ferromagnetic magnetization has been observed from a low concentration of 0.2%Eu. Our µSR experiments have clearly shown evidence of an intrinsic ferromagnetic transition in Sr_0.98_Eu_0.02_Al_2_O_4_.

## 2 Results and Discussion

### 2.1 Crystal Structure and Luminescence Properties

All materials were insulative, and the single crystals were nearly transparent. Eu substituted Sr in the lattice up to ≈3 at%, as previously reported for the similarly prepared polycrystalline samples,^[^ ^20^ ^]^ as well as in single crystals.^[^ ^21^ ^]^ The strong reductive atmosphere of Ar‐5%H_2_ during sintering ensured an Eu^2+^ ionic state in the samples.^[^ ^20^ ^]^ Samples used in the present study contained no impurity phase or aggregation, as suggested by the powder X‐ray diffraction (Figure S1, Supporting Information), the picture of the transparent single crystals and the single‐crystal X‐ray diffraction (Figure S2, Supporting Information). The crystal structures of both Sr_0.998_Eu_0.002_Al_2_O_4_ and Sr_0.98_Eu_0.02_Al_2_O_4_ were determined to be monoclinic *P*2_1_ structures, as summarized in Table S1,2 (Supporting Information), which shows the same crystal structure as that reported for undoped SrAl_2_O_4_.^[^ ^22^ ^]^ In Sr_0.998_Eu_0.002_Al_2_O_4_, an oxygen vacancy of 0.9(±0.3)% was found at one O site (O1 in Table S1 and Figure S3, Supporting Information). Structural inspection revealed that defects occurred at the oxygen site nearest to Sr (Figure S3, Supporting Information), which was relatively loosely bonded to the surrounding Al and Sr, suggesting that the oxygen vacancies were induced largely by the incorporation of Eu into the lattice. Thermoluminescence, as shown in Figure S4 (Supporting Information) suggests that the defects trap electrons in this material, which provides the necessary condition for strong ML.

**Figure 1:**
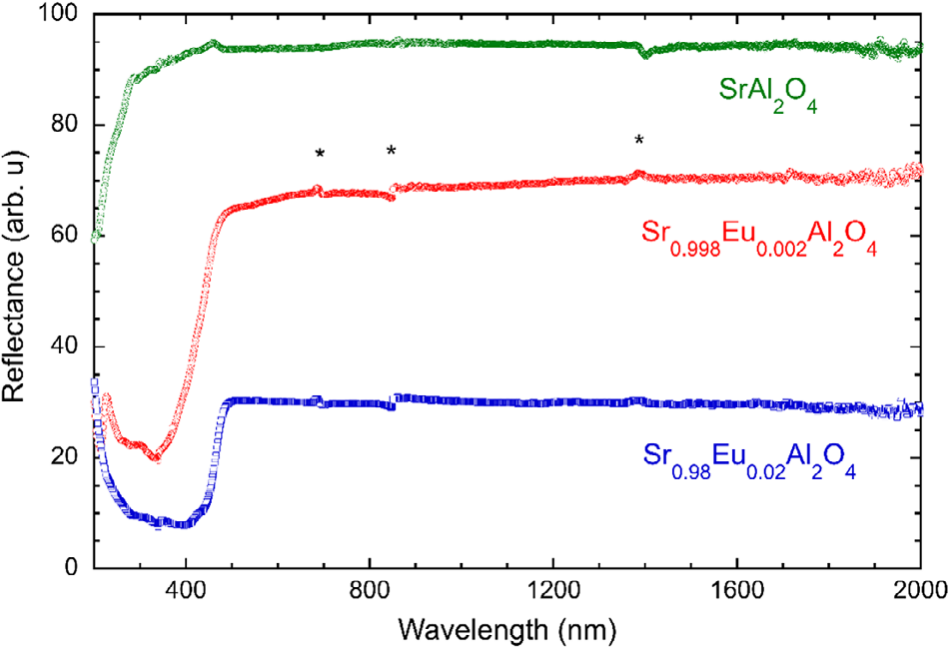
Optical reflectance for undoped SrAl_2_O_4_ and Eu:SrAl_2_O_4_. The wide‐gap semiconductor property of SrAl_2_O_4_ is supported by the band‐gap absorption below λ ∼ 280 nm, corresponding to an *E*_g_ > 4.43 eV in consistency to theoretical calculations.^[^ ^23^, ^24^, ^25^ ^]^ The absorption below  λ ∼ 500 nm in Eu:SrAl_2_O_4_ is due to the excitation of Eu^2+^.^[^ ^26^ ^]^ The small anomaly ≈λ ∼ 460 nm in the spectrum for SrAl_2_O_4_ might be caused by trace impurity of Eu^2+^ in the raw material of SrCO_3_. The small anomalies marked with the asterisks are due to the mismatched background in the UV/VIS/NIR multi‐spectrophotometer system. The different baselines for the two crystals are due to their different size.

Optical reflectance in **Figure** **1** shows that SrAl_2_O_4_ is a semiconductor with a bandgap absorption below *λ *< 280 nm, corresponding to an *E*_g_ > 4.4 eV in consistency to theoretical calculated *E*_g_ = 4.5−6.6 eV.^[^ ^23^, ^24^, ^25^ ^]^ Meanwhile, the absorption at  λ < 500−300 nm in Eu^2+^:SrAl_2_O_4_ should be due to the $4f^{7}\to 4f^{6}5d^{1}$ excitation of Eu^2+^,^[^ ^26^ ^]^ since Eu substitution of Sr has little effect on the band gap.^[^ ^27^ ^]^ This interpretation is directly supported by the photoluminescence described below.

**Figure 2:**
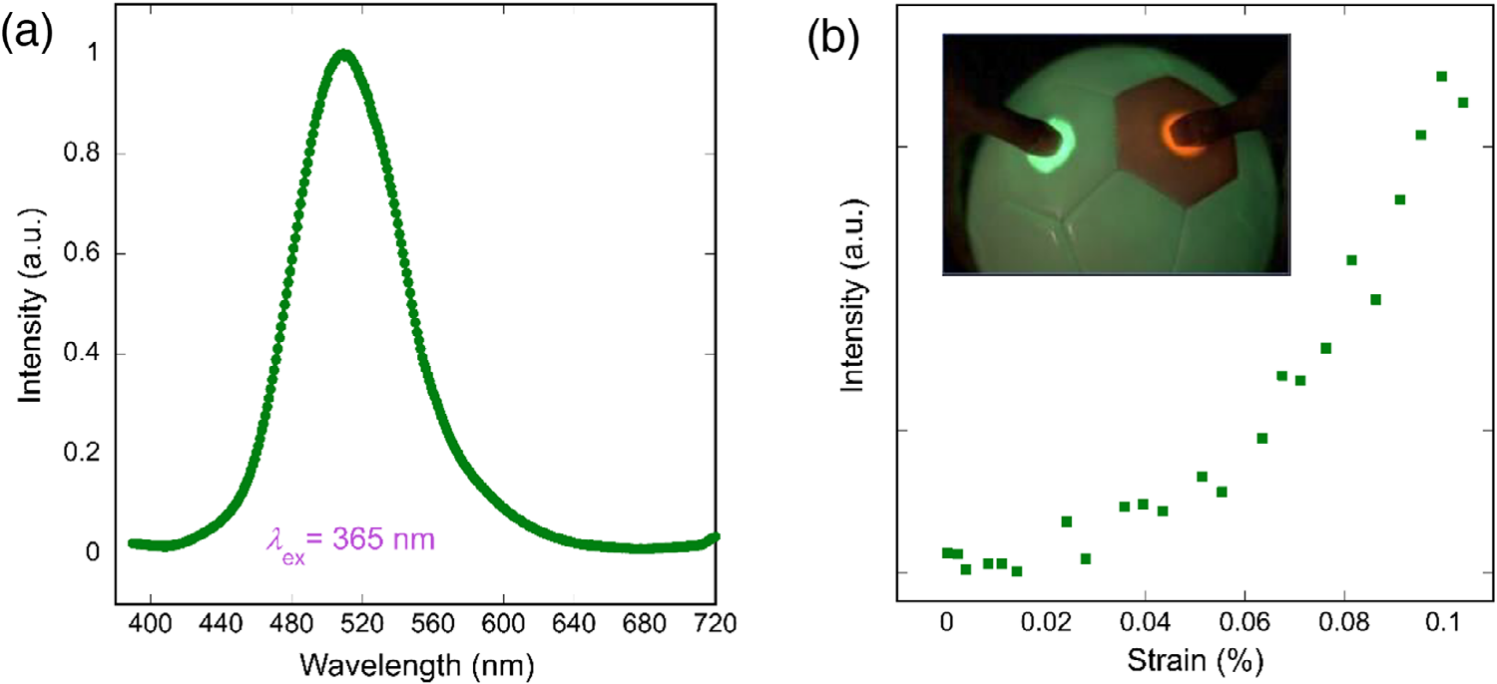
a) Photoluminescence peak centered ≈*λ* ∼ 510 nm in 0.2%Eu^2+^:SrAl_2_O_4_ polycrystalline sample under UV excitation at *λ* = 365 nm, in consistency with the reported $4f^{6}5d^{1}\to 4f^{7}$ transition of Eu^2+^.^[^ ^26^ ^]^ b) Mechanoluminescence response to strain in polycrystalline 0.2%Eu^2+^:SrAl_2_O_4_. The inset picture shows a valley ball coated with 0.2%Eu^2+^:SrAl_2_O_4_ and another ML material Sm^3+^:Sr_3_Sn_2_O_7_,^[^ ^28^ ^]^ emitting green and red light, respectively, upon finger contact.

The Eu^2+^:SrAl_2_O_4_ is a well‐known luminescent material, as illustrated by the photoluminescence and mechanoluminescence in **Figure** **2**. Photoluminescence centered ≈510 nm, which is commonly ascribed to the parity‐allowed electric dipole transition, $4f^{6}5d^{1}\to 4f^{7}$, of the Eu^2+^ion,^[^ ^26^ ^]^ can be produced under UV excitation. Strain due to a mechanical force can induce intense green ML in this material, therefore, it can be used as a visible sensor for the strain, as shown by the inset picture in Figure 2b. Furthermore, this property can be used in various applications to detect strain distribution and potential micro‐cracks in infrastructures by dispersing or painting the ML material to infrastructures (detailed demonstration in Supporting Videos). SrAl_2_O_4_ co‐doped with Eu^2+^ and Dy^3+^ are also known as the most studied long‐lasting phosphor for storage applications such as scintillators and dosimeters that utilize its merit of transparency.^[^ ^29^ ^]^

### 2.2 Unexpected Ferromagnetic Magnetization and Relation to Polaron

**Figure 3:**
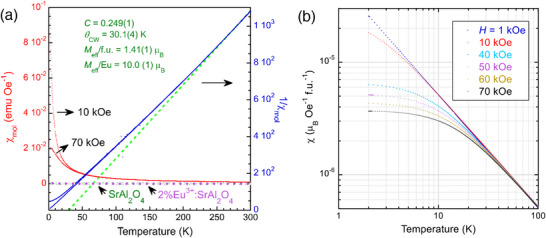
a) Magnetic susceptibility (*H* = 10 kOe, 70 kOe, respectively) for polycrystalline Sr_0.98_Eu_0.02_Al_2_O_4_. The dashed line represents a Curie‐Weiss fit $\chi =\frac{C}{T-\theta_{\mathrm{CW}}}$ with *C* = 0.249(1), *θ*_CW_ = 30.1(4) K. Susceptibilities of nonmagnetic SrAl_2_O_4_ and 2%Eu^3+^:SrAl_2_O_4_ are also plotted for comparison. b) Susceptibilities under various external fields showing nonlinear *M‐* *H* magnetization below ≈80 K.

Undoped SrAl_2_O_4_ and 2%Eu^3+^:SrAl_2_O_4_, which were obtained with sintering in air, are nonmagnetic with an exceedingly small negative magnetic susceptibility as plotted in the bottom of **Figure** **3**a. On the contrast, Eu^2+^:SrAl_2_O_4_ samples are magnetic. The weak‐field magnetization under zero‐field cooling and field‐cooling modes shows no difference, excluding the possibility of spin glass in the present system. The magnetic susceptibility of polycrystalline Sr_0.98_Eu_0.02_Al_2_O_4_ shows a Curie−Weiss temperature *θ*_CW_ = 30.1(4) K, implying ferromagnetic interactions (Figure 3a). The Curie−Weiss constant *C* = 0.249(1) corresponds to an effective moment of 1.41(1) *µ*_B_ per formula Sr_0.98_Eu_0.02_Al_2_O_4_ and 10.0 (1) *µ*_B_ per Eu, which is unreasonably large compared to the small fraction of Eu^2+^ if only Eu^2+^ carries the magnetic moment.

**Figure 4:**
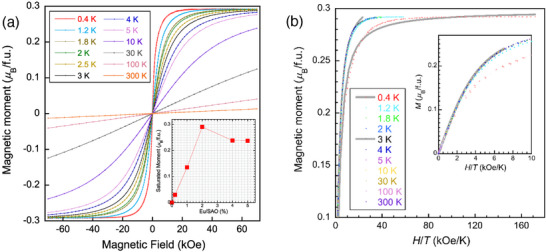
a) Magnetization *vs* field, *M*–*H*, in polycrystalline Sr_0.98_Eu_0.02_Al_2_O_4_. The inset plot shows the saturated magnetic moment at 2 K for samples doped with 0−5% Eu. b) *M*‐*H*/*T* plots showing deviation from a paramagnetic Brillouin function fit (the gray thick lines). The inset plot is an enlarged *M*‐*H*/*T* view, and the gray line is Brillouin fit for the 10 K data.

As seen from the susceptibilities under various magnetic fields in Figure 3b, nonlinear magnetization appears below ≈80 K. The magnetization vs field, *M*–*H*, shows unexpected ferromagnetic behavior below ≈3 K (**Figure** **4**a), with a saturated magnetic moment of ≈14 *µ*_B_/Eu. This is surprisingly large compared to the theoretical 7.93 *µ*_B_ for isolated Eu^2+^ ion, suggesting a substantial contribution from the lattice. Negligible hysteresis in *M‐* *H* was observed, which was different from that of a conventional ferromagnet. However, its absence can be reasonably explained by considering the sparsely distributed concentration of Eu^2+^ in the lattice, which would lead to an extraordinarily large single domain size causing negligible *M‐* *H* hysteresis if a ferromagnetic phase is formed in this material. The inset plot shows that the substitution limit in Sr_1‐_ *_x_* Eu*_x_* Al_2_O_4_ is approximately higher than *x* = 0.02, in consistency with previous reports.^[^ ^20^, ^21^ ^]^ At the same time, it clearly demonstrates that the magnetism originates from the Eu^2+^ incorporated into the lattice. The *M*‐*H*/*T* plots in Figure 4b show a prominent deviation from the paramagnetic Brillouin function fit for magnetizations below ≈3 K. The low‐field magnetization at 0.4 K is much larger than the paramagnetic fit, indicating a ferromagnetic picture.

**Figure 5:**
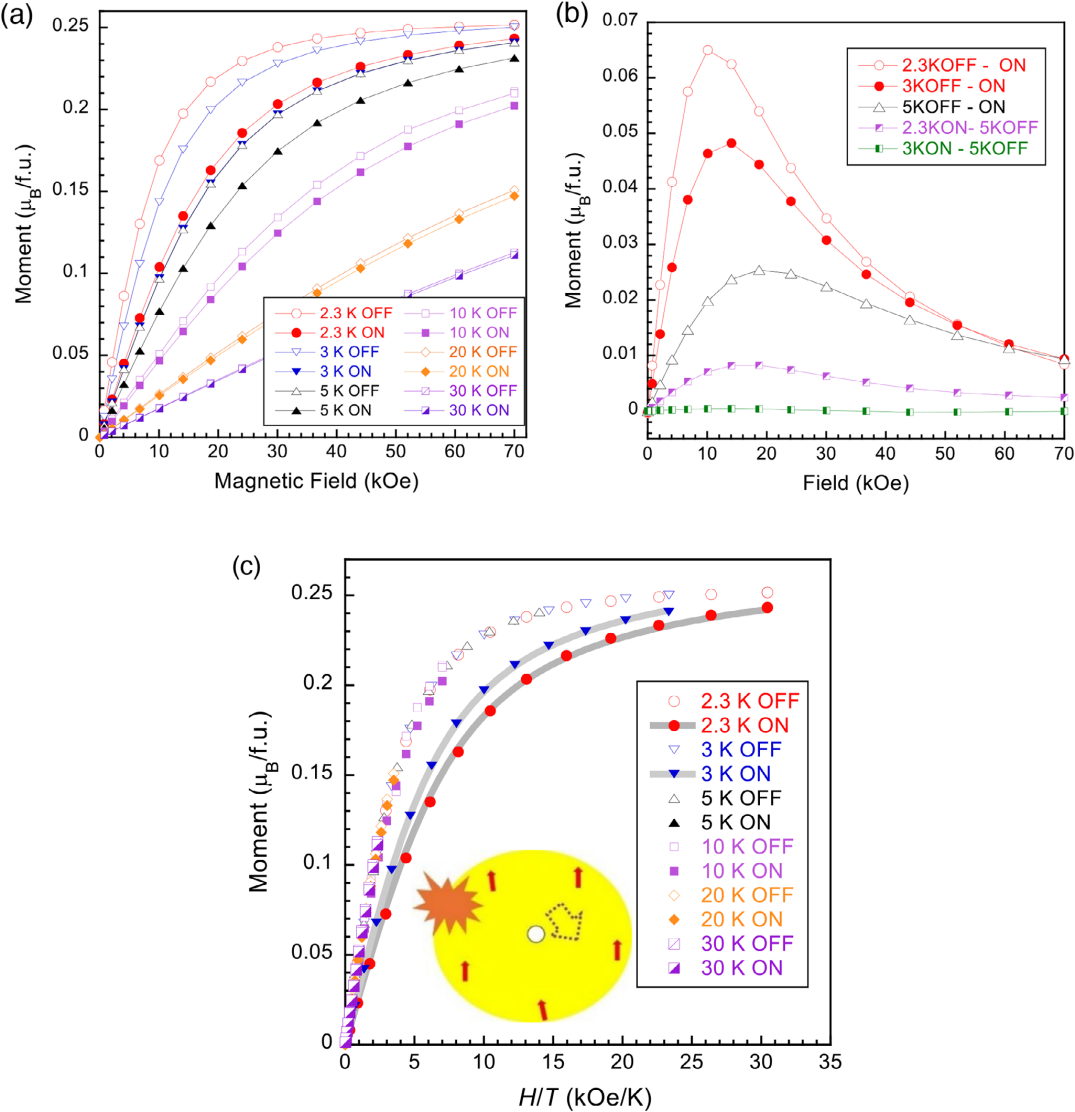
a) *M*–*H* magnetization curves for polycrystalline Sr_0.98_Eu_0.02_Al_2_O_4_ under light radiation from a LED light of color temperature 3100 K (incident light parallel to the magnetic field direction). b) The magnetization difference due to light radiation. c) *M*‐*H*/*T* plot. The thick gray curves are fitted with Brillouin function, showing the transformation to superparamagnetic‐like state by light radiation. The inset figure illustrates the excitation of a bound magnetic polaron from an oxygen vacancy upon light radiation, wherein the dashed‐lined arrow denotes the polaron spin and thin arrows the Eu^2+^ spins.

Because direct magnetic interactions cannot be expected in the present system, in which the magnetic ions of Eu^2+^ are sparsely distributed, magnetic coupling via an intermediary should exist. Previous theoretical studies have suggested that the bound magnetic polaron, a bubble of spins ordered ferromagnetically by exchange interaction with an effective mass carrier in a localized state, is the most suitable model for possible diluted ferromagnetism.^[^ ^19^, ^30^ ^]^ The feature of electrons trapped in oxygen vacancies in the present material, as seen in the thermoluminescence spectrum in Figure S4 (Supporting Information), and its excellent ML properties makes it a candidate for a bound magnetic polaron. To test this model, we first compared the magnetization *vs* temperature data referring to the BMP percolation theory in ref. [30] and found it incompatible since it predicted a small contribution of localized holes (electrons) to the total sample magnetization, which contradicts with the observed excessive moment. We further measured the magnetization under light radiation to test the BMP scenario. A prominent light‐radiation effect was observed below 30 K (**Figure** **5**a), which is more clearly seen by plotting their difference in Figure 5b. The light radiation effect decreased quickly with increasing temperature, suggesting that it is strongly correlated to the magnetic state. Meanwhile, it is noted that the 2.3K‐ON (light radiated at 2.3 K) data in Figure 5a approached the 5K‐OFF (without light radiation at 5 K) data, and the 3K‐ON data almost overlapped with the 5K‐OFF data. Although the light‐radiation raised the stablizable temperature for measurement to 2.3 K, we have carefully confirmed that the above‐mentioned approaching and overlapping are not caused due to an heating effect (this is readily understandable since the temperature was stable during the measuremnt that involved movement of a 4‐cm scan through two coils in the equipment and the environmental exchange gas flow of He ensured a quick thermal equilibrium). The *M*‐*H*/*T* plots in Figure 5c show a distinct transformation to the paramagnetic state by light radiation. In view of the long‐wavelength light of the LED, the excitation of bound polarons should be the reason for this transformation. As a comparable reference for the bound polaron, absorption peaks at *λ* > 2000 nm have been reported for Eu‐rich EuO, where the bounded electrons to oxygen vacancies were reported to produce bound magnetic polarons and long‐wavelength absorption.^[^ ^31^ ^]^ The presence of bound magnetic polarons can explain well the approaching behaviors of the 2.3K‐ON and 3K‐ON data to those of 5K‐OFF in Figure 5a,b, in which the light‐radiation at 2.3 and 3 K would excite the bound magnetic polarons, transforming the system to a superparamagnetic‐like state and making their magnetization close to the nearly paramagnetic one at 5 K. The large saturated magnetic moment of ≈14 *µ*_B_/Eu as revealed in Figure 4a is also in consistency with the BMP,^[^ ^32^ ^]^ which explains the excessive magnetic moments. In this model, the spins of magnetic ions would be aligned by an external magnetic field of ≈15 kOe and those of vacancy‐trapped electrons aligned by weaker fields. The peak feature of the light radiation effect in Figure 5b agrees well with this model.

### 2.3 Magnetic Transition Witnessed by µSR

**Figure 6:**
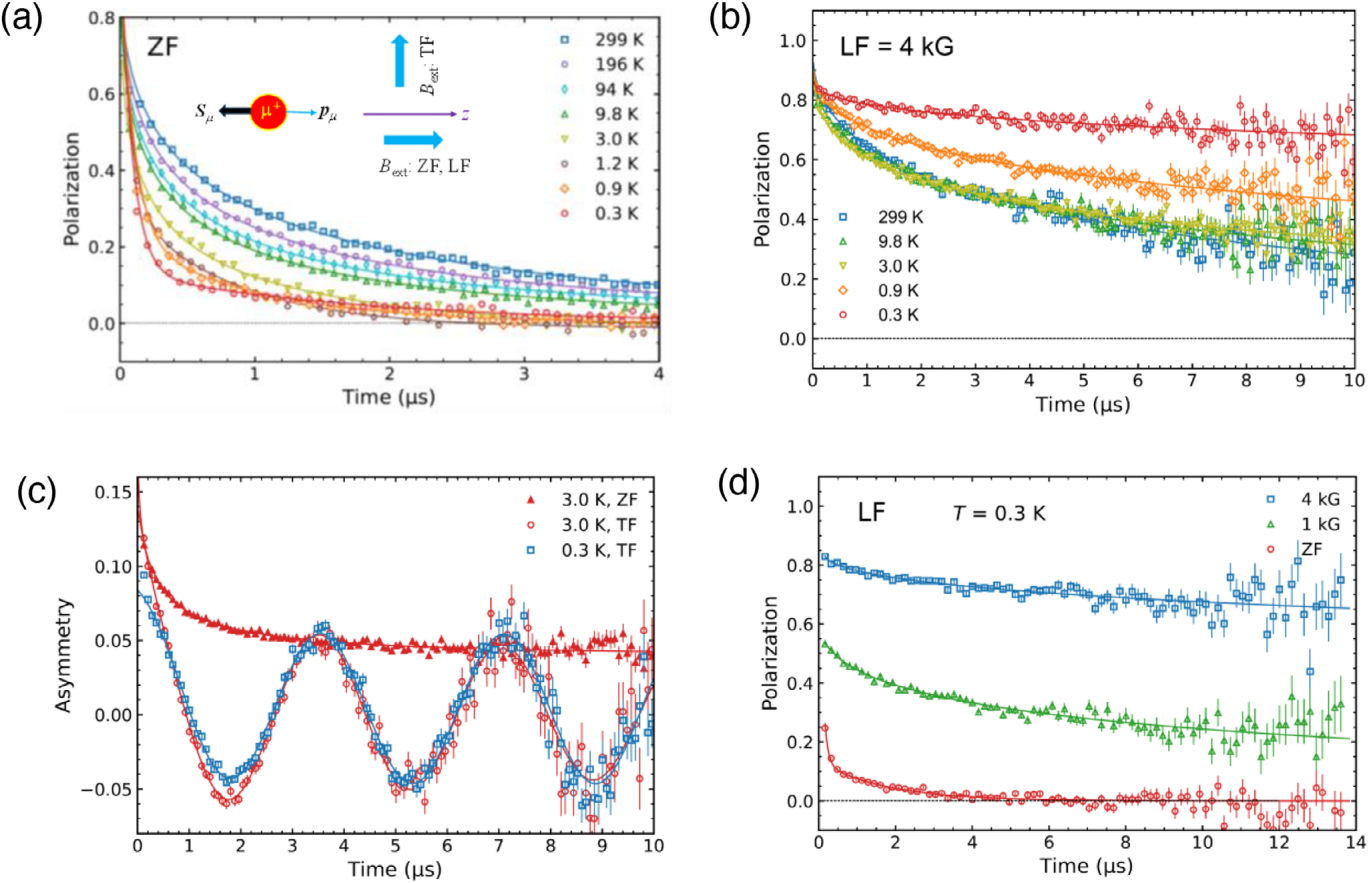
a,b) ZF and LF‐µSR spectra for polycrystalline Sr_0.98_Eu_0.02_Al_2_O_4_ at typical temperatures. The inset illustration in (a) shows µSR experiments in the three modes of ZF, LF, and TF‐µSR. c) Comparison of TF‐µSR at 3 K and 0.3 K. The ZF spectra at 3 K are also plotted for an easy comprehension of the TF spectra. d) LF‐µSR at 0.3 K showing the presence of a static spontaneous field in the material. Solid lines in all plots are fitted curves as described in the text.

The spin freezing and magnetic transition in Sr_0.98_Eu_0.02_Al_2_O_4_ have been unambiguously demonstrated by µSR. µSR is known to provide reliable bulk information on the specimen^[^ ^33^ ^]^ because the muon spin polarization reflects the volume fraction of the magnetic phase in the specimen, therefore, extrinsic effects due to impurity contamination can be safely excluded. As illustrated in the inset illustration in **Figure** **6**a, the zero field (ZF), longitudinal field (LF) and transverse field (TF) µSR spectra were recorded to study the microscopic magnetic state, where ZF‐µSR probes the unmagnetized ground state in zero external field (wherein the earth field was carefully cancelled to zero), and TF‐ and LF‐µSR under an external magnetic field provide multiple pieces of evidence for the magnetic phase in the sample and its nature.

Muon spin depolarization owing to the internal magnetic field arising from the magnetic ions was observed at high temperatures (Figure 6a). The LF‐µSR spectra in Figure 6b for an external LF of 4 kOe clearly demonstrate the occurrence of a static spontaneous internal field below ≈3 K indicating a magnetic transition. The transition can be also observed in the TF‐µSR spectra in Figure 6c, wherein the uplifted center line of the TF oscillation spectra suggests obviously enhanced internal field upon cooling from 3 to 0.3 K. From the LF decoupling behavior in Figure 6d, the static internal field at the muon stopping site is estimated to be 670−950 G.

**Figure 7:**
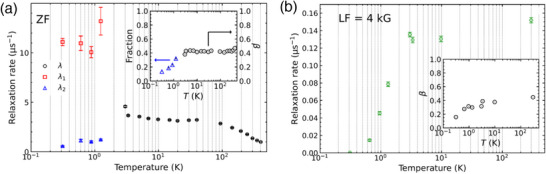
a) Fitted parameters for Sr_0.98_Eu_0.02_Al_2_O_4_ from the ZF‐µSR spectra, and b) LF‐µSR spectra. The inset plot in (a) presents the fitted parameters of *β *and the fraction of slow depolarization component *F*_slow_ as described in the text.

The development of magnetic coupling and the transition into the ordered state can be seen more clearly in the analyzed results as shown in **Figure** **7**a. The ZF‐µSR polarization spectra from high temperature to 3 K and LF‐µSR polarization can be well fitted by $P_{z}\left(t\right)=P_{z}\left(t=0\right)e^{-{\left(\lambda t\right)}^{\beta }}$, where in *β*≠1 implies a distribution of the internal field for the muon spin depolarization. The distribution can be readily interpreted because the implanted muons stop near the oxygen sites in this compound, and there are 200 oxygen sites (i.e., muon stopping sites) per Eu in Sr_0.98_Eu_0.02_Al_2_O_4_. The nonlinear *M‐* *H* behavior below ≈80 K in Figure 3b is supported by the change in the depolarization rate *λ* below 94 K (Figure 7a), suggesting that the nonlinear *M*‐*H* behavior is produced by a change in magnetic coupling. Below 3 K, the ZF‐µSR spectra can no longer be fitted by the stretched exponential function; instead, it must be replaced by the sum of two exponential functions, i.e., $P_{Z}\left(t\right)=F_{slow}e^{-\lambda_{1}t}+F_{fast}e^{-\lambda_{2}t}$. The change in the magnetic state below 3 K is also observed in the depolarization rate *λ *for the LF‐µSR plotted in Figure 7b. The slow and fast depolarizations correspond to the longitudinal and transverse depolarizations, respectively, in a magnetically ordered polycrystalline specimen, wherein spin–lattice relaxation causes longitudinal depolarization and additional spin−spin relaxation causes faster transverse depolarization.^[^ ^34^ ^]^ The µSR polarization is proportional to the volume ratio of the magnetic phase in the specimen; therefore, it reveals the bulk property of the material. Therefore, dilute ferromagnetism is verified intrinsic in the present material.

Notably, the magnetic transition occurs at a temperature far below the occurrence of prominent magnetic coupling, that is, from ≈80 K. This is unusual for magnetic systems outside frustrated quantum spin systems. This can be attributed to the relatively low concentration of polarons in the present material, Eu*_x_* Sr_1‐_ *_x_* Al_2_O_4_, wherein the number of polarons should be limited by the concentration of oxygen vacancies. Therefore, significantly higher *T*_C_ can be expected by effectively enhancing the concentration of polarons, such as by substituting metal ions with magnetic ions of different valences.

## 3 Conclusion

An unconventional superlong‐range magnetic coupling and ferromagnetic spin freezing have been found in a mechanoluminescent material Eu*_x_* Sr_1‐_ *_x_* Al_2_O_4_ (*x* = 0.2−2%), wherein Eu is sparsely incorporated into the lattice to substitute Sr. Experimental results have suggested a bound magnetic‐polaron mechanism responsible for the magnetic coupling and ferromagnetic transition. This finding can be viewed as the first evidence of intrinsic dilute ferromagnetism, challenging the well‐established concept that magnetic transitions in nonmetals require the presence of a considerably large proportion of magnetic spins in the system. Whereas traditional electronics are based on the control of charge carriers doped into semiconductors, the intrinsic dilute ferromagnetism due to doped spins allows the control of quantum spin states for spintronics applications. The proposed system is a promising mechanoluminescent material. The discovery of dilute ferromagnetism in mechanoluminescent semiconductors with magnetic ions simultaneously serving as emitting centers for mechanoluminescence upon the application of a mechanical force has important implications in unprecedented force‐light‐spin‐electron multiple conversion and control for future applications. Although the present dilute ferromagnetism and the resulting light radiation effect occur at low temperatures, high‐*T*_C_ can be achieved by increasing the polaron density. Such an effort is in progress with the expected *T*_C_ enhancement obtained.

## 4 Experimental Section

### Materials Synthesis

Polycrystalline Eu:SrAl_2_O_4_ was prepared by solid‐state reaction of mixed and pressed pellets of fine powders of high‐purity SrCO_3_, Al_2_O_3,_ and Eu_2_O_3_ in stoichiometric ratios with 1 wt.% H_3_BO_3_ in Al_2_O_3_ crucibles at 1350 °C for 4 h in Ar‐5%H_2_ atmosphere after calcination at 800 °C in air for 1 h, wherein the H_3_BO_3_ served as a flux evaporating at high temperatures and the Ar‐5%H_2_ reductive atmosphere ensured the Eu^2+^ ionic state in the samples. Single crystals were grown from the polycrystalline samples in Rod‐shape in an Ar atmosphere using a floating‐zone infrared gold image furnace.

### Measurements

Single‐crystal synchrotron X‐ray diffraction measurements were performed on single crystals of Sr_0.998_Eu_0.002_Al_2_O_4_ and Sr_0.98_Eu_0.02_Al_2_O_4_. The experiment for Sr_0.998_Eu_0.002_Al_2_O_4_ was carried out at BL02B1 beamline of SPring‐8 synchrotron facility using wavelength of 0.2589 Å. The beamline was equipped with Pilatus 3 X CdTe 1M detector. 20576 reflections were collected at 100K, in *θ* = 0.879°–14.971° and resolution *d* = 0.50 Å. Measurement for Sr_0.98_Eu_0.02_Al_2_O_4_ was performed at BL41XU beamline of SPring‐8 synchrotron facility using wavelength of 0.700 Å. The beamline was equipped with an EIGER 16M detector. 2420 reflections were collected at 100 K in *θ* = 3.295°–30.791° and resolution *d* = 0.68 Å. Data reduction and absorption correction were performed using the CrysAlisPro software.^[^ ^35^ ^]^ All crystal structures were solved and refined using the SHELXT,^[^ ^36^ ^]^ SHELXL^[^ ^37^ ^]^ and Olex2 software.^[^ ^38^ ^]^ Optical reflectance was measured using a UV/VIS/NIR spectrophotometer (JASCO V‐570). The luminescence was measured under ultraviolet (UV) light using a fluorescence spectrophotometer (JASCO FP8600), and mechanoluminescence was measured using a previously reported system.^[^ ^14^, ^15^, ^16^ ^]^ Magnetization measurements were performed using a commercial SQUID setup (Quantum Design MPMS‐3) in 4 cm scan with additional measurements down to 0.4 K using ^3^He for cooling. Furthermore, magnetization measurements under visible light irradiation were performed using a self‐made sample rod for the SQUID, to which an LED light with a color temperature of 3100 K (Schott Megalight 100) was introduced through optical fibers (CK‐10E0.25, Mitsubishi Electric). µSR experiments were conducted at the S1 beamline of the muon facility at J‐PARC using a polarized positive surface muon beam. Liquids ^4^He and ^3^He were each used for cooling in the two related experiments. The polycrystalline samples for µSR were ground and thoroughly mixed with thin varnish (GE7031, AXIS Co.) to ensure good thermal contact.

## Conflict of Interest

The authors declare no conflict of interest.

## Author Contributions

C.N. X. contributed equally to this work. X.G.Z. and C.N.X. performed conceptualization; C.N.X. performed material synthesis; X.G.Z. and I.Y. performed magnetization and muon spin spectroscopy experiments; E.N. and T.G. performed crystal structure determination; J.N. and A.K. supported to muon spectroscopy experiment; T.K. performed fabrication of light‐radiation equipment and support to magnetization measurements; X.G.Z. Wrote the final manuscript.

## Supporting information



Supporting Information

Supplemental Movie 1

Supplemental Movie 2

## Data Availability

The data that support the findings of this study are available from the corresponding author upon reasonable request.
