# Supplementary material for: Superlong‐Range Magnetic Coupling and Ferromagnetic Spin Freezing in Mechanoluminescent Semiconductor Eu:SrAl2O4
Source: Adv Sci (Weinh). 2025 Jul 31;12(38):e09474. doi: 10.1002/advs.202509474 (PMC12520534; doi:10.1002/advs.202509474)
Supplement: Supplementary file 1 — Supporting Information [file ADVS-12-e09474-s003.docx]

Supporting Information

Superlong-range magnetic coupling and ferromagnetic spin freezing in mechanoluminescent semiconductor Eu:SrAl_2_O_4_

Xu-Guang Zheng^1,2*^, Ichihiro Yamauchi^1^, Tomasz Galica^3^, Eiji Nishibori^3^, Tatsuya Kawa^4^, Jumpei G. Nakamura^5^, Akihiro Koda^5^, Chao-Nan Xu^2†^

**Contents:**

Two supplementary tables showing detailed information on the crystal structures, four figure illustrating sample quality, bonding environment and the electron trapping in the crystal as **Supporting Materials**. Two demonstration videos are also provided for the present mechanoluminescent material emitting green light upon applying a mechanical force, and application for detecting early-time fatigue cracks in infrastructures.

**Table S1**. Structure information for Sr_0.998_Eu_0.002_Al_2_O_4_ at 100 K, which was solved and refined from collected reflections of 20576 (6264 unique) with Goodness of Fit on *F*^2^ = 1.036; *R*_int_ = 0.0410; *R*_1_ = 0.0238; *S* = 1.034; wR_2_ (all data) = 0.0643.

**Table S2**. Structure information for Sr_0.98_Eu_0.02_Al_2_O_4_ at 293(2) K. 2420 (1515 unique) with Goodness of Fit on *F*^2^ = 1.254; *R*_int_ = 0.0270; *R*_1_ = 0.0713; *S* = 1.253; wR_2_ (all data) = 0.2387.

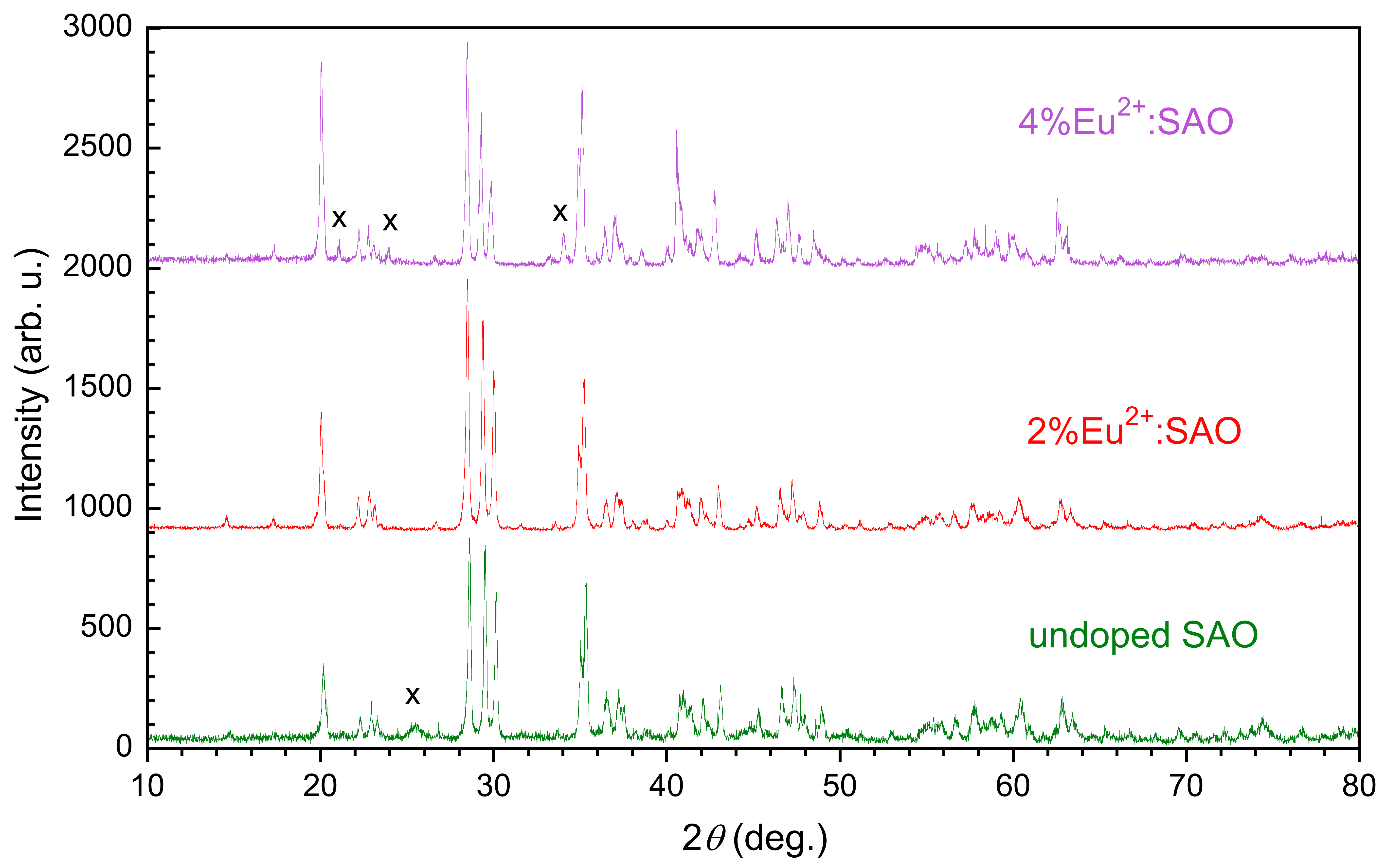


**Figure S1.** Powder X-ray diffraction patterns for undoped SrAl_2_O_4_ (SAO), and SAO doped with 2%Eu^2+^ and 4%Eu^2+^. The symbol x denotes impurity phase. The substitution by Eu improves the crystallinity with no impurity phase upto 2% Eu^2+^. In the 4%Eu:SAO sample, which exceeds the substitution limit in SrAl_2_O_4_, impurity phase began to develope.


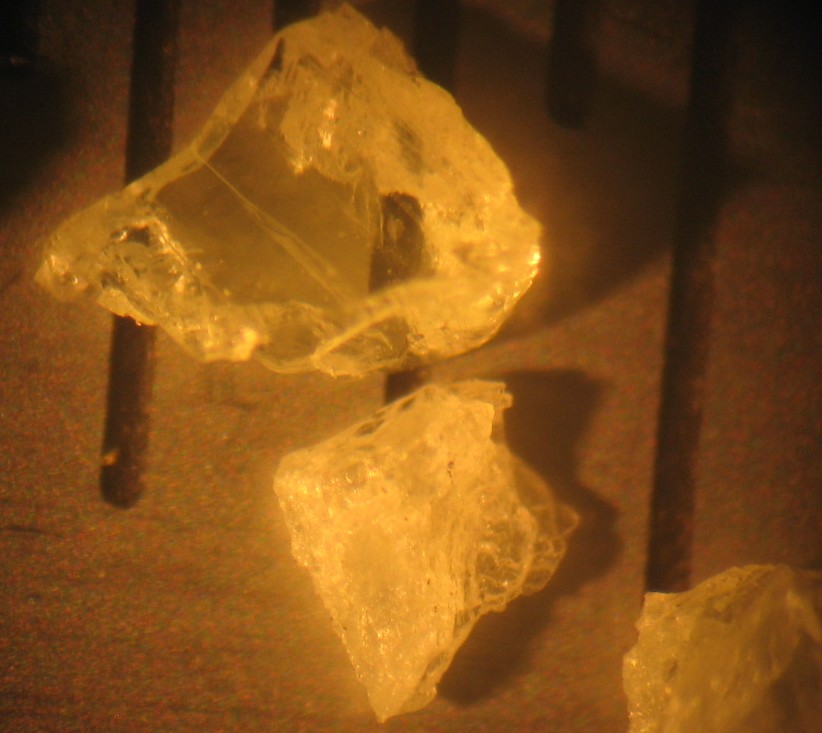


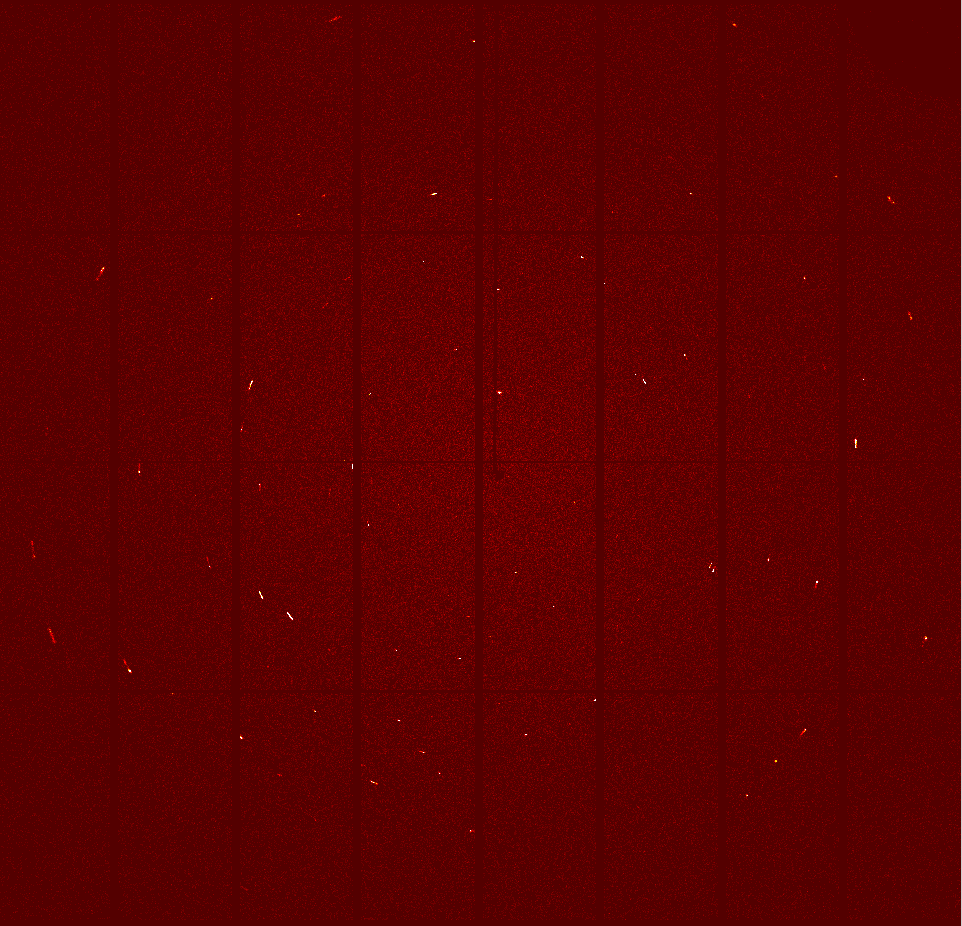


**Figure S2**. Upper panel: optical picture of Sr_0.98_Eu_0.02_Al_2_O_4_ crystals sized in ~ 1 cm^3^. Lower panel: an example of syncrotron X-ray diffraction patterns for single-crystal Sr_0.98_Eu_0.02_Al_2_O_4_, wherein all diffraction spots are indexable with monoclinic Sr_0.98_Eu_0.02_Al_2_O_4_. Twins and multi domains exist extensively in the single crystal.


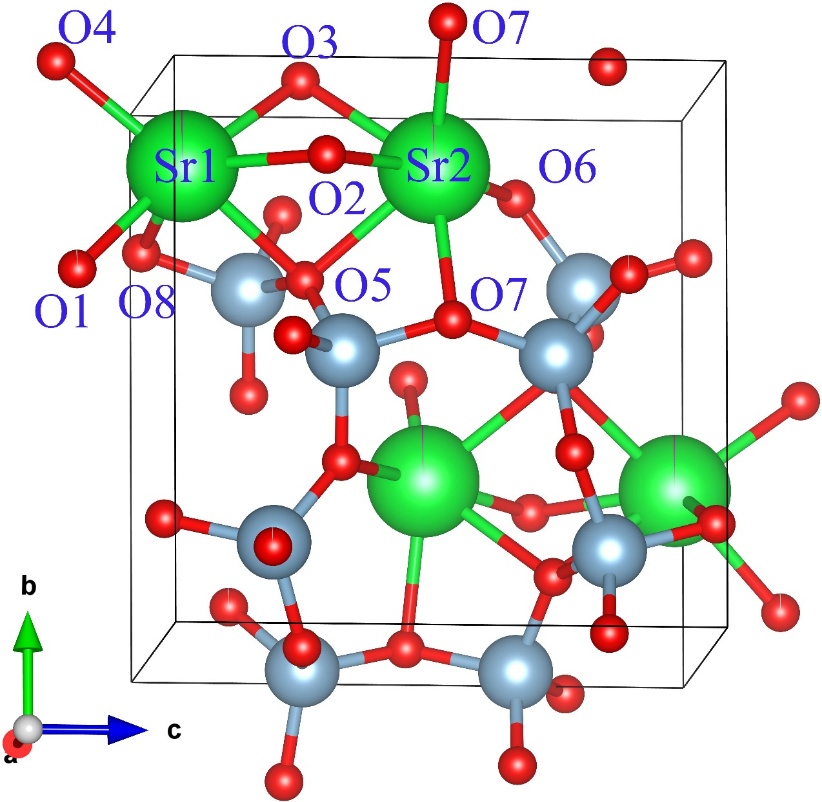

**Figure S3.** Upper panel: crystal structure of Sr_0.998_Eu_0.002_Al_2_O_4_ displayed using software VESTA.^[33]^ Green, red and gray balls represent Sr, O, Al atoms, respectively. Lower panel: Bond length of oxygen with metal ions in the crystal structure. The O1 site, where vacancies exist, is relatively loosely bonded to surrounding Al and Sr.


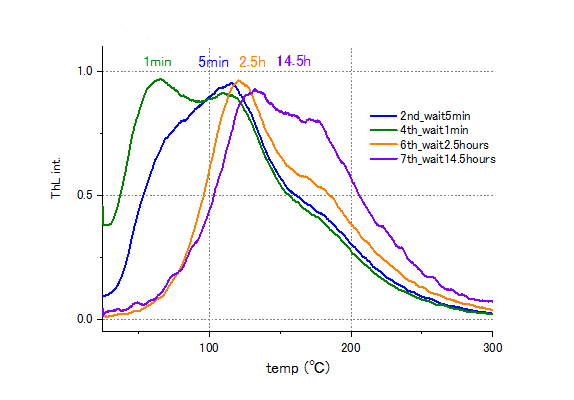


**Figure S4.** Thermoluminescence in Sr_0.998_Eu_0.002_Al_2_O_4_ with waiting time from 1 min to 14.5 h after excitation by UV light. The peaks around 120 and 170 ℃ suggest luminescence from trapped electrons.
